# A lentiviral system for efficient knockdown of proteins in neuronal cultures [version 1; referees: 2 approved]

**DOI:** 10.12688/mniopenres.12766.1

**Published:** 2017-12-05

**Authors:** Brigitte Ritter, Shawn M. Ferguson, Pietro De Camilli, Peter S. McPherson

**Affiliations:** 1 Department of Biochemistry, Boston University School of Medicine, Boston, MA, 02118, USA; 2 Department of Neurology and Neurosurgery, Montreal Neurological Institute, McGill University, Montreal, H3A 2B4, Canada; 3 Department of Cell Biology, Program in Cellular Neuroscience, Neurodegeneration and Repair, Department of Cell Biology, Yale University School of Medicine, New Haven, CT, 06510, USA; 4 Department of Cell Biology, Department of Neuroscience, Kavli Institute for Neurosciences, Howard Hughes Medical Institute, Yale University School of Medicine, New Haven, CT, 06510, USA

## Abstract

We have devised a protocol for highly efficient and specific knockdown of proteins in neuronal cultures. Small hairpin RNAs (shRNAs) are embedded into a microRNA (miRNA) context by oligo annealing to create shRNAmiRs, which are expressed from within the 3′-UTR of a reporter protein. This reporter protein/synthetic miRNA cassette is transferred to a targeting vector and lentivirus is produced in HEK-293-T cells following co-transfection of the targeting vector with three additional vectors encoding essential lentiviral proteins. Mature virus is harvested by collecting culture medium from transfected HEK-293-T cells, the virus is purified by centrifugation, and virus titers are determined prior to addition to neuronal cultures. Near 100% transduction efficiency of cultured hippocampal neurons is routinely observed and allows for the population-wide inhibition of target protein expression and the simultaneous knockdown of multiple proteins with little or no toxicity. The lentivirus generated can be used for protein knockdown in multiple neuronal culture models and at a variety of developmental stages. The steps from shRNAmiR design to ready-to-use virus stocks can be completed in as little as two weeks.

## 1. Background and results

RNA interference (RNAi) is one of the most important tools in biomedical research. Traditionally, RNAi has been accomplished using one of two approaches. In the first, synthetic small inhibitory RNA (siRNA) species are synthesized and transfected directly into cultured cells, generally using lipofection. Alternatively, small hairpin RNAs (shRNAs) are generated in plasmid vectors under the control of polymerase (pol) III promoters such as H1 or R6. These plasmids are transfected into cells where the expressed shRNA is processed into a siRNA species in the cytoplasm via the actions of the nuclease dicer (Brummelkamp *et al*., 2002; Paddison *et al*., 2002). More recently, RNAi design has used synthetic shRNAs in the structural context of microRNAs (shRNAmiRs) (Stegmeier *et al*., 2005). Like shRNAs, shRNAmiRs have a stem-loop structure where the stem is composed of annealed sense and antisense strands that are perfectly matched to the target mRNA. However, the stem-loop structure is embedded into a microRNA context and these structures can be driven downstream of pol II promoters such as CMV (Stegmeier *et al*., 2005).

When expressed in cells, shRNAmiRs are processed by the endogenous miRNA processing machinery, specifically, they are first processed in the nucleus by the enzyme Drosha, liberating a shRNA that is subsequently processed by Dicer (Hammond, 2005). It is thought that engagement of the endogenous miRNA machinery allows for more efficient processing and knockdown of mRNA. Importantly, shRNAmiRs also appear to have less toxicity when compared to shRNAs. For example, targeting mutant Huntingtin disease (HD) transgenes in mouse models of HD with RNAi has effectively reduced neuropathological and behavioral deficits (Harper *et al*., 2005; Rodriguez-Lebron *et al*., 2005). Unexpectedly however, shRNAs were found to induce neurotoxicity in the striatum, even when the shRNA had mismatches with the huntingtin target (McBride *et al*., 2008). The same sequences in the shRNAmiR context were as efficient in knockdown, but did not display the toxicity of the shRNAs when injected into the striatum (McBride *et al*., 2008). A further advantage of the shRNAmiR system is that reporter genes can be incorporated into the shRNAmiR transcript allowing for easy detection of expressing cells (Stegmeier *et al*., 2005). Thus, shRNAmiRs have many advantages and yet as for siRNAs and shRNAs, the knockdown reagents must be delivered into cells by transfection. As a results, these approaches have major limitations, especially for long-term studies and the use of post-mitotic cells such as neurons (Sariyer, 2013).

Viral vectors have been used for efficient transfer of protein-encoding genes into a wide variety of cells and have become a popular tool for the introduction of RNAi molecules. The technology has been developed to the point that barcoded, lentiviral-based shRNA libraries that target the entire human genome have been generated (Silva *et al*., 2005) and used for genome wide loss-of-function screens (Luo *et al*., 2009). A major advantage of lentiviral particles is that they can be used in post-mitotic cells and protocols for the lentiviral-delivery of shRNAs into neurons have been described (Harper & Gonzalez-Alegre, 2008). Lentivirus has also been used for the delivery of shRNAmiRs (Stegmeier *et al*., 2005) and Nielsen *et al*. (2009) demonstrated the ability of lentiviral-driven shRNAmiRs to knockdown expression of reporter constructs in neurons with no reported toxicity. Thus, combining shRNAmiRs with lentiviral delivery appears to provide an efficient and non-toxic RNAi method that can target a wide variety of cells, including post-mitotic primary neurons.

**Figure 1:**
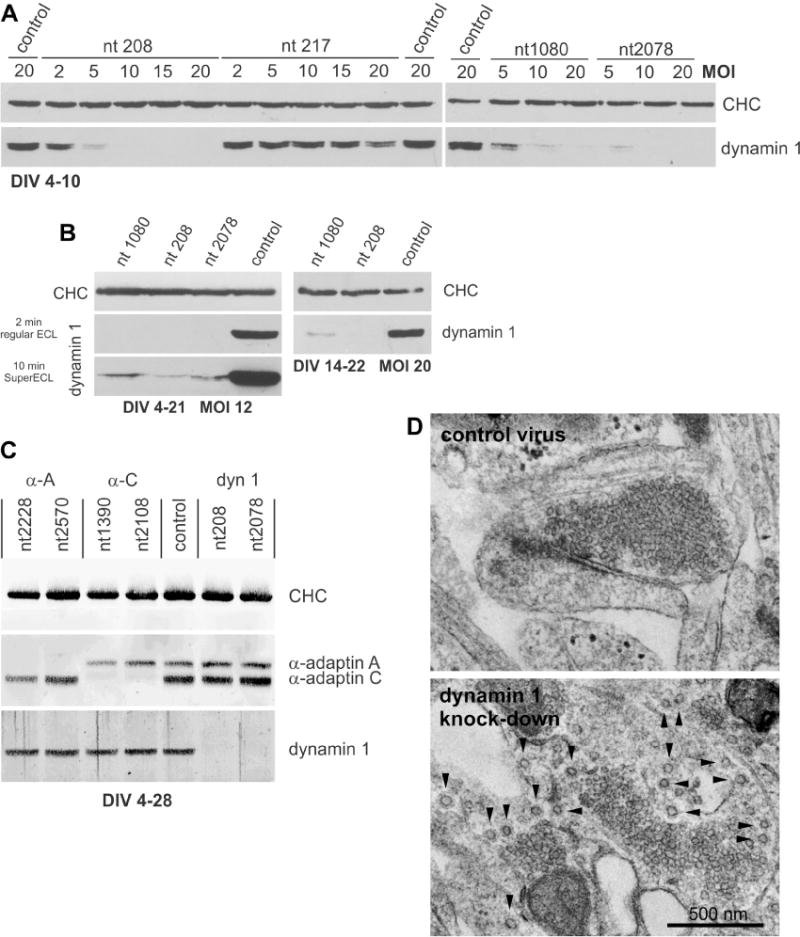
Lentiviral-mediated expression of shRNAmiR knockdown cassettes allows for efficient protein knockdown in primary hippocampal neurons (**A**–**C**) Western blot analysis of total protein extracts from rat primary hippocampal neurons for various proteins as indicated. Clathrin heavy chain (CHC) was used as loading control. dyn 1: dynamin 1, α-A: α-adaptinA, α-C: α-adaptinC, control: shRNAmiR-control virus. (**A**) Primary hippocampal neurons were transduced on DIV 4 with different shRNAmiR viruses directed against dynamin 1 as indicated by the nt positions and a range of MOIs were tested for each virus. Cells were harvested for analysis on DIV 10. The generation of four to five different knockdown viruses against one target protein usually yields in at least two viruses with high knockdown efficacy. (**B**) Primary hippocampal neurons were transduced on DIV 4 (left) or DIV 14 (right) with different shRNAmiR viruses and directed against dynamin 1 and MOIs as indicated and Western blot for dynamin 1 confirms efficient protein knockdown in neurons transduced as different stages of *in vitro* differentiation. The dynamin 1 blot on the left also demonstrated the efficacy of the KD. Dynamin 1 is virtually undetectable in knockdown neurons after ECL exposure for two minutes using regular ECL and faint traces of residual dynamin 1 are only detected under conditions that super-saturate the signal detected in shRNAmiR-control neurons (10 min super-ECL). (**C**) Knockdown of additional proteins involved in clathrin-mediated endocytosis as indicated. Neurons were transduced at DIV 4 and harvested for analysis on DIV 28, demonstrating the persistent knockdown of target proteins over extended periods of time. (**D**) Electron microscopy analysis of mouse primary cortical neurons transduced with control and dynamin 1 knockdown shRNAmiR viruses as indicated. The lentivirus-mediated knockdown recapitulates the phenotype seen in neurons from dynamin 1 knockout animals and arrow highlight the abundance of clathrin-coated pits that accumulate due to dynamin 1 depletion.

**Figure 2:**
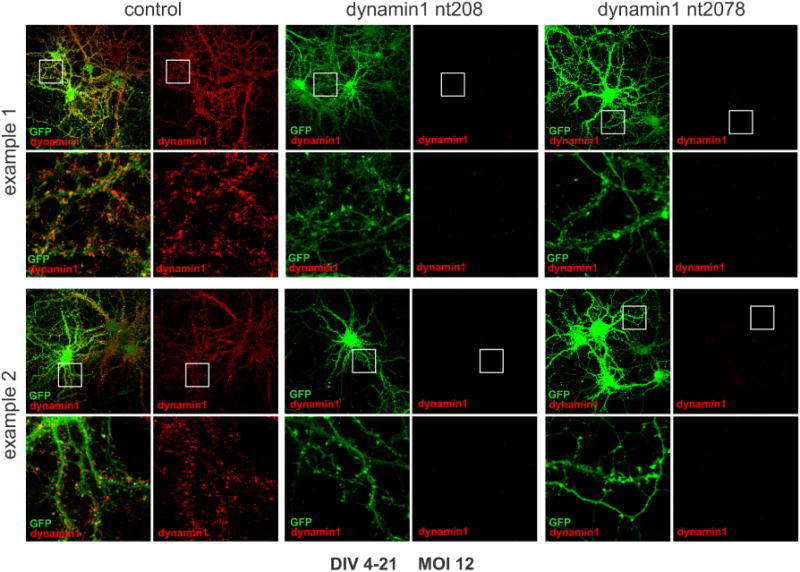
Immunofluorescence analysis of dynamin 1 expression in control and dynamin 1 knockdown neurons Primary rat hippocampal neurons were transduced on DIV 4 with control or dynamin 1 knockdown shRNAmiR viruses as indicated using an MOI of 12 and processed for immunofluorescence on DIV 21. Two examples for each condition are shown. GFP is the fluorescent marker protein expressed as part of the shRNAmiR cassette and dynamin 1 was detected using an antibody directed against endogenous dynamin 1. Dynamin 1 is readily detected in neurons transduced with the control virus but is virtually undetectable in neurons transduced with the knockdown viruses. The areas in the white boxes seen in the lower magnification images is shown at higher magnification below each image.

We have developed a lentiviral system driving shRNAmiRs that is highly efficient for the knockdown of specific target proteins in neurons and other cell types. The shRNAmiR is embedded in the 3’UTR of emerald green fluorescent protein (EmGFP) or monomeric red fluorescent protein (mRFP) downstream of a CMV promoter. The system has been used extensively in hippocampal neurons cultured from rat brain. We routinely achieve near 100% transduction efficiencies, which can decrease the expression of target proteins to levels not detectable by Western blot (Katoh *et al*., 2009; Thomas *et al*., 2009) (Figure 1A–C) and immunofluorescence imaging (Figure 2). We generally generate four or five shRNAmiR viruses with non-overlapping knockdown sequences for each target protein to find at least two viruses with high knockdown efficiency. For example, for the neuronal protein dynamin 1, a GTPase critical for recycling of synaptic vesicles in the presynapse (Ferguson *et al*., 2007), we generated 4 separate shRNAmiR sequences indicated by the first nucleotide in the sequence (nt 208, nt 217, nt 1080 and nt 2078) of which three gave efficient knockdown (Figure 1A and B). When examining the multiplicity of infection (MOI) suitable for knockdown we have found that a MOI as low as 2 can lead to detectable knockdown with efficient depletion generally achieved at a MOI of 5 (Figure 1A and B). This low MOI allows for the opportunity to combine viruses for simultaneous double knockdown. For example, we have used this approach in our study of clavesin 1 and 2, two neuron-specific phospholipid- and clathrin-binding proteins (Katoh *et al*., 2009). Viral transduction with shRNAmiRs specific for each isoform at an MOI of 5 allows for knockdown of individual isoforms and combining the two viruses for a total MOI of 10 leads to efficient double knockdown (Katoh *et al*., 2009). shRNAmiR-encoding lentiviruses can also be used to determine the expression of specific protein isoforms. For example, shRNAmiRs targeting the alpha or alphaC isoforms of the AP-2 protein complex involved in endocytosis leads to selective knockdown of the appropriate isoform (Figure 1C). Finally, this system allows one to recapitulate phenotypes caused by gene knockout. For example, analysis of cortical neurons in culture from dynamin 1 knockout mice reveals striking accumulations of clathrin-coated pits that remain trapped on the plasma membrane (Ferguson *et al*., 2007). Notably, lentiviral-mediated knockdown of dynamin 1 in mouse cortical neurons recapitulates this morphological phenotype very accurately (Figure 1D).

An additional benefit of our lentiviral system is the expression of a fluorescent marker in transduced cells that can be used to confirm expression of the shRNAmiR cassette on a single-cell level (Figure 2). Moreover, we are able to transduce primary neurons at various stages of *in vitro* differentiation (Figure 1), which is a prerequisite for studies focused on individual aspects of neuronal function such as neuronal development, synapse formation, or synaptic transmission in mature neurons. Notably, a single round of transduction is sufficient to maintain protein knockdown for several weeks thanks to the stable integration of the shRNAmiR cassette into the cell genome (Figure 1B and C). In addition, we have used lentiviral delivery of shRNAmiRs for efficient protein knockdown in other primary neuronal culture models and a wide variety of mammalian cell lines from different species (Allaire *et al*., 2010; Ceni *et al*., 2010; Chamberland *et al*., 2016; Ritter *et al*., 2013). Thus, our lentiviral system provides an effective and versatile tool to study protein function.

## 2. Materials

### 2.1. Buffers and chemicals

water:autoclaved double-deionized water or commercial ultrapure DNase/RNase-Free distilled water2× annealing buffer:20 mM Tris pH 7.8100 mM NaCl0.2 mM EDTA1× TE buffer:10 mM Tris pH 8.01 mM EDTAspectinomycin:set up 10 mg/ml stock in water and filter sterilize, aliquot and store at −20°Campicillin:set up 100 mg/ml stock in water and filter sterilize, aliquot and store at −20°CLB medium:25 g/l pre-mixed LB medium dissolve in double-deionized water and autoclave to sterilizeLB plates:25 g/l LB medium and 15 g/l agar in double-deionized water- add a stir bar and autoclave to dissolve agar and sterilize the solution- cool down while spinning at low speed on a magnetic stir plate- once the solution has cooled down to touch, add sterile-filtered antibiotics, e.g. 50 μg/ml spectinomycin or 200 μg/ml ampicillin, before pouring plates2M CaCl_2_:dissolve in double-deionized water and filter-sterilize, store at room temperature0.1× TE:1 mM Tris pH 8.00.1 mM EDTAdissolve in double-deionized water, filter-sterilize, aliquot and store at −20°C2× HBS:dissolve 32.8 g NaCl, 0.42 g Na2HPO4, and 23.8 g HEPES in double-deionized water- adjust pH to 6.98 and bring to final volume of 2 l- set 500 ml aside and adjust the pH of the remaining solution to 7.00- set 500 ml aside and adjust the pH of the remaining solution to 7.02- set 500 ml aside and adjust the pH of the remaining solution to 7.04- filter-sterilize all four solutions, aliquot and store at −20°CTo identify the pH/solution with the highest transfection efficiency- plate 5 × 10^6^ HEK-293-T cells each in four 10 cm dishes- the next day, prepare four 1.7 ml microfuge tubes each with  660 μl 0.1× TE,  90 μl 2 M CaCl2,  20 μg targeting vector (EmGFP or mRFP)  10 μg pMDLg/pRRE,  6 μg pMD2.g, and  5 μg pRSV-Rev- mix in one of the four HBS solution into each tube, vigorously pipet up and down to mix- incubate 20 min at room temperature- add transfection solutions to cells and return cells to incubator- the next day, choose the HBS solutions with best transfection efficiency based on the percentage of fluorescent cells in the culture, fluorescence intensity and cell viability.*Note: cells and medium contain virus*.*We usually find that one or two of the four HBS solutions perform significantly better than the others and we test every new batch of HBS solutions made. Only use HBS solutions with the highest transfection efficiency (at least 80+% of EmGFP- or mRFP-positive cells) for virus production (see section 6.1)*.

### 2.2. Oligos and miRNA design

#### 2.2.1. Sequencing and PCR primers (in 5′ to 3′ direction)

pcDNA 6.2 antisense oligoCCTCTAGATCAACCACTTTGTmiRNA-GFP sense BglIIGCGCAGATCTACCGGTCGCCACCATGGTGAGCAAGGGCGAGGAGCmiRNA-RFP sense BglIIGCGCAGATCTACCGGTCGCCACCATGGCCTCCTCCGAGGACGTCmiRNA-XFP antisense XhoIGCGCCTCGAGTGCGGCCGGATCTGGGCCATTTGTTCCATGTGAGTGC

#### 2.2.2. miRNA design

We use the ThermoFisher Scientific BLOCK-iT RNAi Designer (https://rnaidesigner.thermofisher.com/rnaiexpress/) to select target sequences. Make sure to select the miRNA tab from the Target Design Options (Step 2 on the web form). We usually design the target sequences within the open-reading frame (pre-selected option) and stay within the pre-selected 35–55% GC content (step 4 on the web form). On occasion, we have designed target sequences directed against the UTR’s and those resulted in efficient knockdown as well. Make sure to select the correct species such that the software automatically excludes sequences with predicted off-targets hits (step 3, see section 6.2).

We usually select four to five non-overlapping knockdown sequences for each target protein from the highest ranked sequences and try to cover a range of different GC content percentages. The complete miRNA oligo sequences, including linker, miRNA stem sequences and overhangs for cloning, are designed by the software. For each miRNA, two 64-nucleotide oligos are designed and standard purity oligos (desalted and lyophilized) can be ordered from a multitude of commercial providers.

## 3. Protocols - cloning

### 3.1. Anneal oligos

- Resuspend oligos in autoclaved or water to a final concentration of 1 μg/μl (~ 50 μM).

- Mix2.5 μl sense oligo2.5 μl antisense oligo25.0 μl 2× annealing buffer20.0 μl water- Heat 5 min at 95°C in a dry bath, remove metal block and let block/samples cool down on the bench (to about 40°C). Alternatively, the annealing can be done in a thermo cycler.

### 3.2. Phosphorylate oligos

*Make sure to store phosphorylated oligos on ice when in use or at −20°C for long-term storage*.

- Mix1 μl annealed oligos1 μl 10× T4 DNA ligase buffer (NEB)7 μl water1 μl T4 PNK (polynucleotide kinase, NEB)(if using PNK buffer, the reaction needs to be supplemented with ATP)- Incubate 30 min at 37°C for the oligo phosphorylation, then 10 min at 70°C to inactivate the enzyme.- The phosphorylated oligos are now at 5 μM.- Dilute an aliquot, e.g. 5 μl, 1:125 with water to a final conc. of 40 nM.

### 3.3. Digest first vector

*This step is best done in parallel with the oligo annealing/phosphorylation to optimize timing*.

There are two versions of the first vector, pcDNA6.2/GW-EmGFP-miR and pcDNA6.2/GW-mRFP-miR, which only differ by the fluorescent marker being expressed as part of the shRNAmiR cassette.

- Digest 2.5 μg of vector in 50 μl total volume. Add appropriate restriction buffer and 20 units BsaI (e.g. BsaI-HF, NEB). Incubate for 4 hrs at the appropriate temperature.
- Add 1 unit SAP (shrimp alkaline phosphatase, e.g. from Affymetrix) into the restriction mix
- Incubate 1 hr at 37°C for the vector dephosphorylation, then 15 min at 65°C to inactivate the enzyme.
- Add 75 μl 1× TE (final volume in tube 125 μl) to bring vector DNA to 20 ng/μl

*Note: This digest will remove one PvuII restriction site, which is located between the two BsaI sites that flank the oligo insertion site*.

### 3.4. Ligate phosphorylated miRNA oligos into BsaI-digested, dephosphorylated vector

- Mix1 μl BsaI-cut vector (20 ng/μl)1 μl phosphorylated oligos (40 nM)1 μl 10× T4 DNA ligase buffer (NEB)1 μl T4 DNA ligase (400 units/μl, NEB)6 μl water- Ligate at least 1 hr (or over-night) at room temperature

### 3.5. Transformation into bacteria

- We use chemically competent bacteria and the use of recombination-deficient strains, such as DB3.1 or Stable, is recommended. Follow the manufacturer’s recommendations if using commercially-sourced bacteria.
- Plate the transformation reactions on LB plates containing 50 μg/ml spectinomycin and grow bacteria over night at 37°C

### 3.6. Grow over-night cultures

We usually pick 3-4 individual colonies for each construct to have at least two positive clones (based on restriction digest in step 3.7)

- Pick individual colonies and inoculate 2 ml LB containing 50 μg/ml spectinomycin in ventilated tubes.
- Grow in a shaking incubator over night at 37°C and 220 rpm.

### 3.7. DNA isolation and digest

- Isolate the plasmid DNA from the individual bacteria cultures using standard preparation methods.
- Digest an aliquot of the plasmid DNA with PvuII and separate the DNA fragments by agarose gel electrophoresis.
*Note: The BsaI digest of the vector (step 3.3) removes one PvuII restriction site*.
- Expected band patterns:
pcDNA6.2/GW-EmGFP-miR vector:
  positive clones: 4583 bp, 1116 bp
  negative clones: 4100 bp, 1116 bp, 480 bp
pcDNA6.2/GW-mRFP-miR vector:
  positive clones: 3838 bp, 1116 bp, 700 bp
  negative clones: 3838 bp, 1116 bp, 480 bp, 220 bp

Recombination occurs on occasion. Discard any clones with negative or divergent band pattern (see section 6.3).

### 3.8. Sequence verification

Sequence plasmids with the pcDNA 6.2 antisense oligo to confirm absence of mutations in the miRNA oligo insert and correct integration into the vector.

### 3.9. PCR amplification of the expression cassette

To transfer the shRNAmiR expression cassette into the vector for virus production, PCR amplify the expression cassette using the miRNA-XFP antisense XhoI oligo in combination with the miRNA-GFP sense BglII oligo (for pcDNA6.2/GW-EmGFP-miR constructs) or miRNA-RFP sense BglII (for pcDNA6.2/GW-mRFP-miR constructs).

Example PCR mix:5 μl template (10 ng/μl)5 μl 10× ThermoPol buffer (NEB)0.5 μl dNTPs (25 mM)1 μl sense oligo (100 pmol/μl)1 μl antisense oligo (100 pmol/μl)1 μl Vent DNA Polymerase (2 units/μl, NEB)36.5 μl waterExample PCR program:
$\begin{array}{l} \begin{array}{cc} 5\text{min} & 94 \end{array}{}^{\circ}C\\ \begin{array}{ll} 40\text{sec} & 94{}^{\circ}C\\ 40\text{sec} & 52{}^{\circ}C\\ 60\text{sec} & 72{}^{\circ}C \end{array}]35x\\ \begin{array}{ll} 10\text{min} & 72{}^{\circ}C\\ \text{∞} & 4{}^{\circ}C \end{array} \end{array}$

### 3.10. Digest PCR products

- To each PCR reaction, add 20 units BglII, 20 units XhoI, appropriate buffer, and water to a final volume of 100 μl.
- Incubate digests for 2 hrs at 37°C and separate the DNA fragments by agarose gel electrophoresis.
- Cut out approx. 900 bp band and purify DNA from the agarose slab (e.g. using the Qiagen QIAquick Gel Extraction Kit)

### 3.11. Digest virus expression vector pRRLsinPPTeGFP

*This step is best done in parallel with the PCR product digest to optimize timing*.

*The digest removes the eGFP open reading frame from the vector*.

- Digest 1 μg of vector in 50 μl total volume. Add appropriate restriction buffer and 20 units BamHI and 20 units SalI. Incubate for 2 hrs at 37°C.
- Add 1 unit SAP into the restriction mix
- Incubate 1 hr at 37°C for the vector dephosphorylation, then 15 min at 65°C to inactivate the enzyme.

### 3.12. Ligate PCR product into the digested pRRLsinPPT vector

- Mix:1 μl BamHI/SalI-cut vector (20 ng/μl)2 μl isolated PCR fragment1 μl 10× T4 DNA ligase buffer (NEB)1 μl T4 DNA ligase (400 units/μl, NEB)5 μl water- Incubate at least 1 hr (or over-night) at room temperature

### 3.13. Transformation into bacteria

- We use chemically competent bacteria and recommend the use of recombination-deficient strains such as DB3.1 or Stable. Follow the manufacturer’s recommendations if using commercially-sourced bacteria.
- Plate the transformation reactions on LB plates containing 200 μg/ml ampicillin and grow bacteria over night at 37°C

### 3.14. Grow over-night cultures

We usually pick 4-6 individual colonies for each construct to have at least two positive clones (based on restriction digest in step 3.15)

- Pick individual colonies and inoculate 2 ml LB containing 100 μg/ml ampicillin in ventilated tubes.
- Grow in a shaking incubator over night at 37°C and 220 rpm

### 3.15. DNA isolation and digest

- Isolate the plasmid DNA from the individual bacteria cultures using standard preparation methods.
- Digest an aliquot of the plasmid DNA with EcoRI and XbaI and separate the DNA fragments by agarose gel electrophoresis.
  Expected band patterns:
    positive clones: 6.1 kb, 1.5 kb
    negative clones: 6.1 kb, 1.3 kb

Recombination occurs on occasion (see section 6.3). Discard any clones with negative or divergent band pattern.

*For each row on the agarose gel, also digest one sample containing 1 μg pRRLsinPPTeGFP and run side-by-side with the test samples for comparison*.

The pRRLsinPPT-EmGFP (or mRFP)-shRNAmiR vectors will be used to produce the knockdown viruses. We usually include the target name and the nucleotide position of where the targeting sequence matches to the target open reading frame in the plasmid name, e.g. pRRLsinPPT-EmGFP-shR-NAmiR-dynamin1 nt208.

### 3.16. Sequence verification

Sequence plasmids in both directions with the pRRL sense and pRRL antisense oligos to confirm absence of mutations and correct integration of the miRNA expression cassette into the vector. The final constructs are used to express the different miR targeting sequences.

## 4. Protocols – virus production

*Follow institutional guidelines regulating lentivirus work, including waste treatment and disposal, and obtain the required permit approval before beginning the work. Always use appropriate personal protection equipment when working with lentivirus*.

The system described here is a HIV-based, third generation system, meaning that the producer cells are transiently transfected with four plasmids to produce virus, i.e. individual cells need to take up all four plasmids to produce virus. Thus, good DNA quality, high transfection efficiency and good health of the producer cell line are important to ensure a high virus yield. The packaging mix described below will generate VSV-G-pseudotyed lentivirus to give wide tropism.

### 4.1. Prepare high quality DNA preps

The use of high quality DNA preps is highly recommended. We use the Qiagen HiSpeed Plasmid Maxi Kit and usually purify 0.7-1.0 mg of DNA for each bacteria culture. DNA concentrations should be 0.5-2.0 μg/μl and OD_260/280_ should be at least 1.8, but purities of 1.9 or higher are preferred. Prepare sufficient amounts of DNA for the scale and number of viruses being produced (see step 4.2), keeping in mind that the packaging mix will be combined with each targeting plasmid. Also, remember to include a non-targeting shRNAmiR control. We routinely use a control sequence that has been designed by Qiagen and which has no target in mammalian cells. However, the control shRNAmiR engages the same enzymes and processes that mediate RNAi upon expression of the shRNAmiR knockdown cassettes and thus controls for more variables than the empty target vector or a miR cassette lacking an shRNA target sequence.

The plasmids required are:pRSV-Rev (packing mix)pMD2.g (packaging mix, encodes VSV-G)pMDL/pRRE (packaging mix)pRRLsinPPT-EmGFP (or mRFP)-shRNAmiR-controlpRRLsinPPT-EmGFP (or mRFP)-shRNAmiR-targeting sequence

### 4.2. Prepare HEK-293-T cells and reagents

We maintain HEK-293-T cells in DMEM high glucose medium supplemented with 10% iron-supplemented calf serum at 37°C in an 5% CO_2_-containing, water-saturated atmosphere. To improve cell survival during virus production, the culture medium can be supplemented with 1× non-essential amino acids and 1 mM sodium pyruvate (optional).

We usually plate six 15 cm dishes or T175 flasks with 15 × 10^6^ cells/plate for each virus. Since we often produce six viruses at a time, we grow up eight T175 flasks of HEK-293-T cells to confluency (approx. 80 × 10^6^ cells/plate) to have a large enough cell stock when plating cells for virus production. Also, make sure to have enough incubator space for all dishes/flasks available.

A prep of this size requires approx. 3 l of culture medium to expand the cell stock and for media changes during virus collection as detailed below. For each 15 cm plate/T175 dish, the following DNA amounts are needed:

50 μg expression plasmid (shRNAmiR-targeting sequence or control)
25 μg pMDLg/pRRE
15 μg pMD2.g
12.5 μg pRSV-Rev

In our hands, calcium phosphate-mediated transfection provides and efficient and cheap transfection method. For each 15 cm dish/T175 flask, the following amounts are needed:

1.875 ml 2× HBS
0.225 ml 2M CaCl2
1.65 ml 0.1× TE

### 4.3. Virus production protocol and timeline

#### DAY 1

Plate HEK-293-T at a density of 1.2-1.5 × 10^7^ cells/T175 flasks in 25 ml medium. We use six T175 flasks (or 15 cm dishes) per virus and usually produce six viruses at a time (36 flasks/dishes total).

#### DAY 2

We prepare transfection master mixes for the plates/flasks transfected with the same miRNA construct. For each transfection, prepare 2 tubes (A and B). Volumes are for six dishes/flasks per virus and we use 50 ml conical tubes to set up the transfection mixes.

In each A tube, add 11.25 ml 2× HBS
In each B tube, add 9.9 ml 0.1× TE,
  add DNA for the packaging mix and miRNA vector and mix by shaking,
  add 1.35 ml 2M CaCl_2_ and mix by shaking.

Mix tubes A and B using two pipet guns. Insert a small volume pipet into one pipet gun and continuously bubble the HBS buffer by pushing out air. Using the second gun, dropwise add the content of tube B to tube A, keep bubbling until the complete content of tube B has been added. Start a timer after completion of the first transfection mix. Continue mixing any remaining samples.

Allow for DNA/calcium phosphate precipitate to form for 20–25 min at room temperature, mix by shaking the tube and then add 3.75 ml transfection mix to each of the six 15 cm dishes/T175 flasks. Carefully swirl the plates to mix the transfection solution into the cell culture medium. Return the dishes to the incubator and note the time (time of transfection = 0 hrs).

**8 hrs p.t.** (post transfection), remove the medium from the flasks/dishes and add 15ml of fresh culture medium. This volume is just enough to cover the cells, ensure plates are level in the incubator to avoid cells loss due to cells falling dry.

#### DAY 3

Virus-producing cells release virus particles into the medium. *Thus, from here on out, consider that cells and medium contain virus*. Before collecting the first batch of virus-containing supernatant, check cells for expression of the fluorescent marker (EmGFP or mRFP) to ensure efficient transfection. Combine fluorescent and transmitted light to determine the percentage of transfected cells, the intensity of fluorescent marker expression, and changes in cell morphology (see Section 6.1). At least 80% of cells should express the fluorescent marker protein and the brightest cells should start to round up and may begin to detach. If the transfection efficiency is low, virus titers and purity will be too low to be used in most applications, in particular with primary neurons. The best approach is to start the virus production protocol again.

*HEK-293-T cells attach rather weakly to the culture dishes/flasks and virus-producing cells will be in progressively bad shape over time. It is thus important to remove and replace media slowly and without releasing the liquid directly onto the cells. Pipet against the dish/flask side wall instead to limit premature cell loss and elevated levels of cell debris in the virus-containing supernatant*.

**24 hrs p.t.**, collect the supernatant from each plate, add 15 ml of fresh medium, and return plates to the incubator. If you have multiple plates for one construct, pool the supernatants and store them at 4°C. Since the medium collection containers need to be sterile and will have to be decontaminated or discarded after use, we collect and store the virus-containing medium in plug-cap T75 flasks (BD Falcon), which are large enough to combine the medium for each virus prep (three collections from six plates/flasks, approx. 270 ml), and discard the collection bottles as part of the solid virus-containing waste.

**36 hrs p.t.**, repeat the collection step and return the plates to the incubator. For each construct, pool the supernatants with the ones from the 24 hr collection point and store at 4°C.

#### DAY 4

Collect the medium one last time **48 hrs p.t.**, pool with the earlier collections and discard the plates. Filter the supernatants through 0.45 μm PES filters to remove cell debris. We use disposable, sterile 500 ml-sized filter units consisting of bottle-top filters and receiver bottles.

After filtration, the medium can be aliquoted and stored at −80°C or the virus can be concentrated/purified by centrifugation before being stored at −80°C. Be sure that the tubes/bottles used are rated for the low temperature.

### 4.4. Virus concentration

We prepare primary hippocampal neurons from pre-natal embryos and have noticed that the calf-serum present in the virus-containing medium is toxic for our cultures. To remove the serum, we isolate the virus particles from the filtered medium by centrifugation and resuspend the virus-containing pellet in DMEM only.

- Centrifugation set-up will depend on the centrifuges, rotors and size of centrifugation tubes/bottles available. Under any circumstance, do not overfill tubes to prevent virus-containing medium from leaking into the rotor and centrifuge. Depending on the total medium volume, we have successfully used several parameters, including centrifugation at:
54,000 × gfor 2 hrsusing 30 ml tubes7,440 × gfor 16 hrsusing 50 ml tubes16,900 × gfor 10 hrsusing 500 ml tubes

All centrifugations should be performed at 4°C to preserve virus viability.

*We strongly recommend to use centrifuge tubes/bottles made from polycarbonate, which are clear enough to see the pellet, which has a faint beige, translucent color, often with smaller, darker core. Marking the position of the centrifuge tube pointing outward will make it easier to locate the pellet*.

- Following centrifugation, move tubes carefully as to not agitate the pellet. Circle the pellet on the outside of the tube with a marker before pouring off the medium after as the pellet is harder to see without the liquid.
- Pour off the liquid into a container with bleach in one smooth motion. Do not let liquid swap back and forth across the pellet.
- Store the tubes upside down on an absorbent sheet to let any remaining fluid collect away from the pellet.
- Wait approx. 10 min for the pellet to dry, remove any residual medium at the neck of the tube and resuspend the virus pellet in approx. 1/2000 of the volume originally spun in the tube.
- Be sure to wash the complete pellet off the tube wall and avoid introducing bubbles as much as possible. A large portion of the pellet consists of proteins from the medium and may also contain cell debris from the producer cell line. For samples with high concentrations of virus, we sometimes see a slightly more white-looking rim within the pellet that likely corresponds to the majority of the viral particles. However, these rims are very hard to see and the quality of any virus prep should be determined by titration (step 4.5).
- Aliquot the resuspended virus into 0.5 ml screw-cap tubes with O-ring seals and store virus at −80°C.
For example, we resuspend the pellet coming from 270 ml of supernatant in 140 μl DMEM. Usually, we are able to recover approx. 120 μl from the tube, which we store in 10 μl aliquots.

### 4.5. Virus titration

*Freeze/thaw cycles can result in loss of activity of lentiviral particles. We thus recommend to freeze any virus before titration such that all aliquots of a prep will give comparable transduction efficiencies*.

Any given virus will transduce different cell lines with a cell line-specific efficiency, i.e. a titer established using one cell line will most likely not yield the same transduction efficiency on a different cell line. However, using a reference cell line for titration does allow to equalize the amount of virus needed for different viruses used in one experiment as well as across multiple virus preps. We use HEK-293-T cells to determine virus titers.

- Plate HEK-293-T cells at 40,000 cells/well in 24-well plates, 12 wells for each virus to be titered.
- After 12–14 hrs (enough time for the cells to attach and spread but too short to go through cell division), remove the culture medium and add 500 μl/well of a 1:2 dilution series starting with a 1:200 dilution for each virus.
- The following day, add 1 ml of culture medium to each well.
- Three days after transduction, remove the medium from the wells and carefully add enough PBS to cover each well. Using fluorescent and transmitted light microscope, locate the well(s) of the dilution series in which approx. 10–30% of the cells express the fluorescent marker (EmGFP or mRFP) encoded in the shRNAmiR cassette. In this range, a linear decrease in transduction efficiency between wells indicates that most cells are transduced by single virus particles, thus allowing to calculate virus titer without skewing the results due to multiple integration events at higher virus concentrations.
- Using the percentage of transduced cells in the well and the position of said well in the dilution series, calculate the titer of the virus stock. For example, 20% fluorescent cells in the ninth well of the dilution curve indicate in a titer of 819,200 transducing units (TU, aka active virus particles) per microliter (see Section 6.4).

## 5. Protocols – knockdown in primary neurons

There are two key components in the transduction protocol to enhance viability of primary neurons:

1. The virus-containing medium has to be removed three hours after addition of the virus.
2. The virus-containing medium has to be replaced with conditioned medium, not fresh culture medium.

### 5.1. Transduction of primary hippocampal neurons

- Isolate and culture primary neurons following established protocols. For example, we prepare primary hippocampal neurons from E18 rat or mouse embryos following the method by Kaech & Banker, 2006. For microscopy studies, we plate 30,000–40,000 cells/well in 24-well plates on poly-L-lysine-coated coverslips (NeuVitro) in 1 ml medium per well. For biochemical assays such as Western blot analysis, we plate 200,000–300,000 cells/well in poly-L-lysine-coated 6-well plates in 2.5 ml medium.
- On the day of transduction, remove half of the medium from each well/culture vessel and store the conditioned medium in the incubator to keep it warm.
- Pipet the virus into the remaining medium in the well/culture vessel and carefully swirl the dish to mix the virus into the medium, then return the cells to the incubator.
- After three hours, remove the virus-containing medium from the cells and replace it with the pre-warmed, conditioned medium.
- Culture and feed the neurons following your standard protocol until analysis.

### 5.2. When to transduce primary hippocampal neurons

The best time to transduce primary neurons depends on the parameters of each experiment. For example, for neuronal development studies, neurons should be transduced as early as possible. We have successfully transduced neurons as early as day-in-vitro 1 (DIV 1). In contrasts, our attempts to transduce neurons during plating or immediately after the cells attached to the culture dish resulted in extensive cell death. For analysis of synapse development, we often transduce neurons between DIV 3 and DIV 7, and the time needed for complete turn-over of the target protein should guide timing.

We have also transduced neurons on DIV 14 and DIV 21, e.g. when testing for changes in synaptic function after synapses were allowed to form. We have not seen significant differences in overall transduction efficiency (i.e. percentage of transduced neurons in the culture) for the various transduction time point. However, the shRNAmiR cassette is currently expressed under the CMV promotor, the activity of which is partially suppressed in mature neurons. In case this decreases knockdown efficiency for certain targets, the design of the shRNAmiR cassette could be modified to include the CAG promoter instead.

### 5.3. When to analyze cells following transduction

The time needed to achieve efficient protein depletion has to be empirically established for each target proteins and depends on the turn-over of the endogenous proteins. In the vast majority of cases, we have achieved maximal knockdown within 2–5 days following transduction and timing appears to be independent of age of the neuronal culture.

The advantage of viral transduction is the stable integration of the shRNAmiR cassette into the cell genome such that no additional transductions are required for knockdown of targets with slow turnover. For example, we routinely transduce neurons on DIV 4 for analysis of mature neurons on DIV 21 (Figure 1 and Figure 2).

### 5.4. How much virus to use for transduction

*We base MOI on the titer determined by the titration of HEK-293-T cells*.

This parameter also needs to be established empirically and we have achieved maximal knockdown with MOIs ranging from 2 to 20 for various target proteins and viruses. We do not use higher MOIs since we have noticed a decrease in cell viability with MOIs of 30 and higher.

### 5.5. Knockdown analysis

Given that viral transductions allows for the population-wide manipulation of neuronal cultures, we determine knockdown efficiency using protein-based read-outs, such as Western blot (Figure 1) and immunofluorescence imaging (Figure 2). Experimental details are included in Supplementary File 1.

## 6. Troubleshooting and other considerations

### 6.1. Transfection efficiency for virus production

To produce virus, each HEK-293-T cells needs to be transfected with all four viruses required for virus production. The expression of the fluorescent marker protein (EmGFP or mRFP) encoded in the shRNAmiR cassette can be used to check transfection efficiency. At least 80% of all cells in the dish should be positive for the fluorescent marker 24 hrs post transfection, and transfection efficiencies of 90+% are desirable. A large population of brightly fluorescent cells that remain single cells and may begin to round up is an encouraging sign. In contrast, low numbers of fluorescent cells and/or low fluorescent intensity indicated suboptimal transfection. In addition, cell fusion indicates an over-abundance of VSV-G expression and a suboptimal plasmid ratio in the transfected cells. Any of these signs indicate sub-optimal virus production and may be cause to abandon this set of cells.

Note that checking for fluorescent protein expression at later time-points in the protocol will skew the result because virus secreted from producing cells can transduce other cells in the dish, thereby increasing the number of intensity of fluorescent cells over time.

Unfortunately, there are multiple reasons for sub-optimal virus production:

#### - DNA quality

Low DNA concentration or purity, or DNA degradation will impair transfection efficiency, e.g. the formation of white, cloudy precipitates during the 20 min incubation of the transfection solution indicates a contamination with proteins. If we run into any transfection issues, we generally switch to new DNA preps for all plasmids.

#### - Transfection solutions

Minute changes in pH of the 2× HBS solution has a major impact on transfection efficiency and efficiency decreases if the solution is stored at room temperature over extended periods of time. Alternative approaches are the use of other transfection reagents, e.g. polyethylenimine (PEI) or Lipofectamine 2000. For the latter, the dishes/flasks for the producer cells should be coated with poly-L-lysine to prevent excessive cell loss. While these other transfection reagents work well, we have had the highest transfection efficiencies using the calcium phosphate approach described here.

#### - Cell health

Contamination of cells, including mycoplasma, can have strong negative effects on virus production even though cells transfect efficiently. In addition, high passage cells eventually stop yielding high virus titers and should be replaced by lower passage cells. Note that the presence of the large T-antigen is essential for virus production and HEK-293 cells cannot replace HEK-293-T cells.

#### - Collection schedule

We collect media from the producer cells every 12 hrs and as a rough estimate, you will yield 25% of your total titer within the first 24 hrs, 50% between 24 and 36 hrs, and another 25% between 36 and 48 hrs. Some protocols call for virus collection up to 72 hrs but we only retrieve very minimal amounts of virus between 48–72 hours. Also, some protocols call for a single medium collection 48 or 72 hrs after transfection, however, this approach yields lower titers in our hands, likely due to the extended exposure of the viral particles to high temperatures in the incubators. In addition, decreases in medium pH, which can occur over longer times, can also decrease virus activity.

### 6.2. Oligo design

The BLOCK-iT RNAi Designer runs into problems when designing miRNA sequences directed against targets the sequences areas shared between splice variants. The work around is to omit the species selection in step 3. However, the target sequences predicted by the algorithm need to be manually checked for potential off-target sequences, e.g. by NCBI Blast against the respective species. Sequences with mismatches at the 3’ end, and if possible, with less than 12–14 continuously matched stretches of sequence should be selected.

Also, check if the oligos contain recognition sequences for any of the restriction enzymes used for cloning. If so, adjust expected band patterning accordingly.

### 6.3. Unwanted recombination during cloning

Use recombination-deficient strains for the transformation of ligations. If recombination persist, make sure not to go over time with the initial growth time during the transformation step. Lowering the temperature to 30°C during the transformation and to overnight growth at room temperature on the plates can help (growth phase may need to be extended to allow colonies to reach normal size).

### 6.4 Calculating virus titers

To calculate your titer in TU/μl (transforming units = active viral particle μl), you need to know

- the number of cells plated per well (e.g. 40,000)
- the dilution that gave a nice transduction rate (let’s say 1:25,600)
- the percentage of transduced cells (let’s say 35%)

In this example, you would have transduced 14,000 cells (35% of 40,000) using 0.01953125 μl of your concentrated virus (with 500 μl/well total, at 1:200 equal 2.5 μl virus (well 1); 1: 400 is 1.25 μl (well 2), …).

Multiply the number of transduced cells (14,000) with the ratio of 1/0.01953125 = 51.2 (this is the factor to get to a full μl based on the dilution needed to transduce 14,000 cells). Therefore, the titer of this example virus is 716,800 TU/μl (or 720,000 TU/μl).

## Supplementary Material

Supplementary File 1
