# Supplementary material for: A lentiviral system for efficient knockdown of proteins in neuronal cultures [version 1; referees: 2 approved]
Source: MNI Open Res. Author manuscript; Available in PMC 2018 Jan 17. (PMC5771425; doi:10.12688/mniopenres.12766.1)
Supplement: Supplementary File 1 [file NIHMS929929-supplement-Supplementary_File_1.tgz › 0d92f9ea-d1a9-4d62-9da2-c732f9635993.docx]

**Supplementary File 1**

**S1. Materials and methods**

**Microscopy.** For immunofluorescence microscopy, primary rat hippocampal neurons were plated at 40,000 cells per well on 12 mm, poly-L-lysine-coated coverslips, no. 1.5, in 24-well plates in 1 ml of neuronal culture medium per well. On DIV 4, 50% of the medium was removed from each well and pre-warmed in the incubator. Control and dynamin 1 knockdown viruses were added to the neurons at an MOI of 12. After three hours, the virus-containing medium was removed and replaced with the pre-warmed conditioned medium collected prior to transductions. The neurons were fed on DIV 7 and DIV 14. On DIV 21, cells were washed with PBS, fixed with 2% paraformaldehyde in PBS for 10 min at room temperature, and permeabilized for 1 min with 0.2% Triton X-100 in PBS. Following one wash with PBS, cells were incubated for 1 hr at room temperature with an antibody against dynamin 1 (bullwinkle, David *et al.*, 1996) diluted in 0.02% Triton X-100 in PBS. Cells were washed with 0.02% Triton X-100 in PBS and then incubated for 45 min at room temperature with Alexa-Fluor 546-conjugated secondary antibody diluted in 0.02% Triton X-100 in PBS. Cells were washed twice with diluted in 0.02% Triton X-100 in PBS, once with PBS, rinsed in double-distilled water and mounted in Fluorescence Mounting Medium (cat no. S3023, DAKO). Fluorescence images for dynamin 1 and GFP, which is expressed as part of the lentiviral knockdown cassette, were acquired on a ZEISS LSM 710 confocal microscope. For electron microscopy, primary mouse cortical control and dynamin 1 knockdown neurons were processed as previously described (Ferguson *et al.*, 2007).

**Protein extraction and Western blot.** For Western blot analysis of protein expression levels, primary rat hippocampal neurons were plated at 300,000 cells per well in poly-L-lysine-coated 6-well plates. Neurons were transduced at various DIV as indicated following the procedure described for immunofluorescence microscopy. On the day of harvest, neurons were washed once with PBS, lysed in ice-cold lysis buffer (10 mM Hepes, pH 7.4, 1% Triton X-100, 0.83 mM benzamidine, 0.23 mM PMSF, 0.5 µg/ml aprotinin, 0.5 µg/ml leupeptin) and centrifuged for 2 min at 21,000 *g* to remove cell fragments. Equal amounts of total protein were resolved by SDS-PAGE, and expression levels were determined by western blotting. Primary antibodies were directed against dynamin (Hudy 1, cat no. MABT188, Millipore Sigma), clathrin heavy chain (CHC, clone 23, cat no. 610499, BD Biosciences), and α-adaptinA/α-adaptinC (clone 100/2, cat no. A4325, Millipore Sigma). Secondary antibodies were coupled to horseradish peroxidase (HRP) or near-infrared fluorescent dyes for detection by enhanced chemiluminescence (ECL) or fluorescence imaging on an Odyssey Imaging System (LI-COR Biosciences), respectively.

**S2. Supplementary references**

David C, McPherson PS, Mundigl O, de Camilli P. 1996. A role of amphiphysin in synaptic vesicle endocytosis suggested by its binding to dynamin in nerve terminals. Proc Natl Acad Sci U S A. 93:331-5.

Ferguson SM, Brasnjo G, Hayashi M, Wölfel M, Collesi C, Giovedi S, Raimondi A, Gong LW, Ariel P, Paradise S, O'Toole E, Flavell R, Cremona O, Miesenböck G, Ryan TA, De Camilli P. 2007. A selective activity-dependent requirement for dynamin 1 in synaptic vesicle endocytosis. Science. 316:570-4.
